# Resolving Molecular Interactions in Protein Folding Trajectories with NCIPLOT

**DOI:** 10.1021/acs.jcim.5c01501

**Published:** 2025-09-16

**Authors:** Asier Urriolabeitia, Julia Contreras-García, David De Sancho, Xabier López

**Affiliations:** † Polimero eta Material Aurreratuak: Fisika, Kimika eta Teknologia, Kimika Fakultatea, UPV/EHU & Donostia International Physics Center (DIPC), PK 1072, 20018 Donostia-San Sebastian, Euskadi, Spain; ‡ Laboratoire de Chimie Théorique, Sorbonne Université and CNRS, F-75005 Paris, France

## Abstract

Noncovalent interactions (NCIs) are fundamental to the structure, stability, and function of proteins. These interactions form complex networks that control how different protein regions relate to each other or to external molecules (e.g., solvent, ligands, cofactors, other proteins), shaping the energy landscape of the system. Molecular dynamics (MD) simulations are widely used to study proteins and other biomolecules, helping to explore their structures and transitions between them at atomic resolution. However, the analysis of MD trajectories is often limited to the estimation of geometric features or metrics relating instantaneous configurations to reference structures, which may overlook relevant details of the interactions that drive conformational changes. In this work, we propose a systematic approach to the analysis of simulation data based on NCIs. We use electron density features from topologically meaningful regions to characterize inter-residue NCIs, computing them with NCIPLOT4 across MD simulations to investigate their presence, conformational relevance, and temporal evolution. This enables a direct, data-driven view of how specific interactions contribute to the stability and rearrangement of structural elements. This allows us to map the interactions shaping protein conformations and how they change along certain processes. We apply this framework to ultralong equilibrium trajectories of protein folding, revealing patterns of interaction changes that correspond to distinct folding pathways. This NCI-based approach provides a powerful complement to the traditional structural analysis toolbox, deepening our understanding of protein folding dynamics.

## Graphical abstract

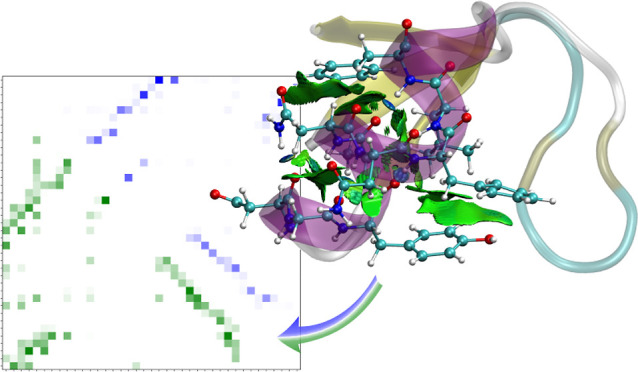

## Introduction

Noncovalent interactions (NCIs) play a pivotal role in the structure of proteins, governing their assembly, stability, and dynamics.^1^ They also underpin functions such as structural support, catalysis, recognition, and transport, which rely on both a defined 3D structure and the ability to switch rapidly between conformations, allowing them to interact with other molecules, respond to environmental changes, and perform complex biological tasks.^2^ Peptide bonds define the protein sequence, while NCIs like hydrogen bonds, π-stacking, and dispersion forces, shape secondary, tertiary, and quaternary structures by promoting favorable interactions between nonadjacent regions.^3^ Despite their modest individual strength, NCIs form vast dynamic networks that allow proteins to adopt multiple conformational states, facilitating processes such as allosteric regulation, ligand binding, and protein–protein interactions.^4,5^

One of the most fundamental processes governed by NCI is protein folding, in which an unstructured polypeptide chain undergoes a spontaneous transition to a well-defined, functional and thermodynamically stable form, its native state.^6,7^ This conformational transition is driven by interactions both within the protein and between the protein and the solvent. Intraprotein interactions promote the formation of secondary structure elements and tertiary contacts, leading to the native structure. Meanwhile, solvent interactions are related to hydrophobic collapse, in which the nonpolar regions of the protein are shielded from the aqueous environment while the polar regions are exposed, ultimately lowering the system’s free energy and stabilizing the folded conformation.^8^ In this context, protein folding can be understood as the process of forming a stable network of NCIs.^9,10^

Molecular dynamics (MD) simulations have become an instrumental tool in the study of proteins by providing atomic-level detail simulations.^11^ This approach faces significant challenges, like sampling relevant conformational transitions between stable states and accurately parametrizing classical force fields that accurately reproduce protein NCIs.^12^ A third problem relates to the analysis of the simulations, which is challenging and burdensome due to the intrinsic high-dimensionality of the resulting data sets (which contain—potentially—the Cartesian coordinates of all protein and solvent atoms with femtosecond resolution).^13,14^ Conventional methods for MD analysis typically focus on purely geometrical features, including interatomic distances, that can be used to define collective coordinates,^15^ often with reference to predefined conformational states. These analysis methods are built into the most commonly used MD simulation packages and have been included in widely adopted libraries such as MDtraj^16^ or MDanalysis.^17^ However, methods that focus on the intricate network of interactions shaping the folding pathway are currently lacking. As a result, traditional approaches may not fully capture the presence, relevance, or dynamic evolution of NCIs throughout folding processes.

The analysis of electron densities provides key features for the identification of NCIs, which appear as real-space regions of low density and low reduced density gradient. Recently, Peccati proposed the use of NCIPLOT4 ^18^ to study biomolecular systems, showcasing its potential through examples involving protein–ligand and protein–protein interactions.^19^ NCIPLOT captures NCIs and distinguishes between strongly attractive, weakly attractive, and strongly repulsive interactions on the basis of the electron density and the sign of the second eigenvalue of its Hessian matrix.^20^ The most recent version, NCIPLOT4, also enables integration of the electron density over these regions, providing a quantitative measure of interaction strength.^18,21^ Peccati’s work focused on interfacial interactions between biomolecules, without resolving which specific regions contributed to the interaction. Moreover, intraprotein interactions, which are central to protein stability, folding and functional dynamics, remain unexplored through this approach. Here, we propose a systematic approach for the application of NCIPLOT4 to the analysis of MD simulations, enabling detailed decomposition of NCIs at the residue level. This allows for the characterization of both intraprotein and interprotein contacts, offering a more comprehensive view of a protein’s interaction landscape.

While in the past, NCIPLOT calculations have been limited to whole biomolecules,^19,22^ in the present workflow we first split the protein into its residues and calculate pairwise density integrals. In this way, the interaction landscape is mapped as a sum of interactions between residue pairs. We present findings obtained through the NCI index to analyze interactions in folding processes in ultralong atomistic simulation trajectories.^23,24^ Unlike traditional analysis methods, our detailed workflow based on NCIPLOT provides a comprehensive description of all noncovalent forces at play throughout the folding process. By mapping these interactions in detail, this methodology allows for deeper insights into how these forces drive the formation and stabilization of protein structure, ultimately enhancing our understanding of the intricate mechanisms underlying protein folding.

## Materials and Methods

In this work we introduce a detailed workflow for the analysis of MD simulation trajectories based on the calculation of density integrals using NCIPLOT (see Figure 1). Although we focus on the specific case of protein folding trajectories obtained with a classical force field as an illustrative example, the procedure is general and can be applied to other types of simulation data sets involving protein–protein interactions or ligand binding with an arbitrary level of theory.

**Figure 1:**
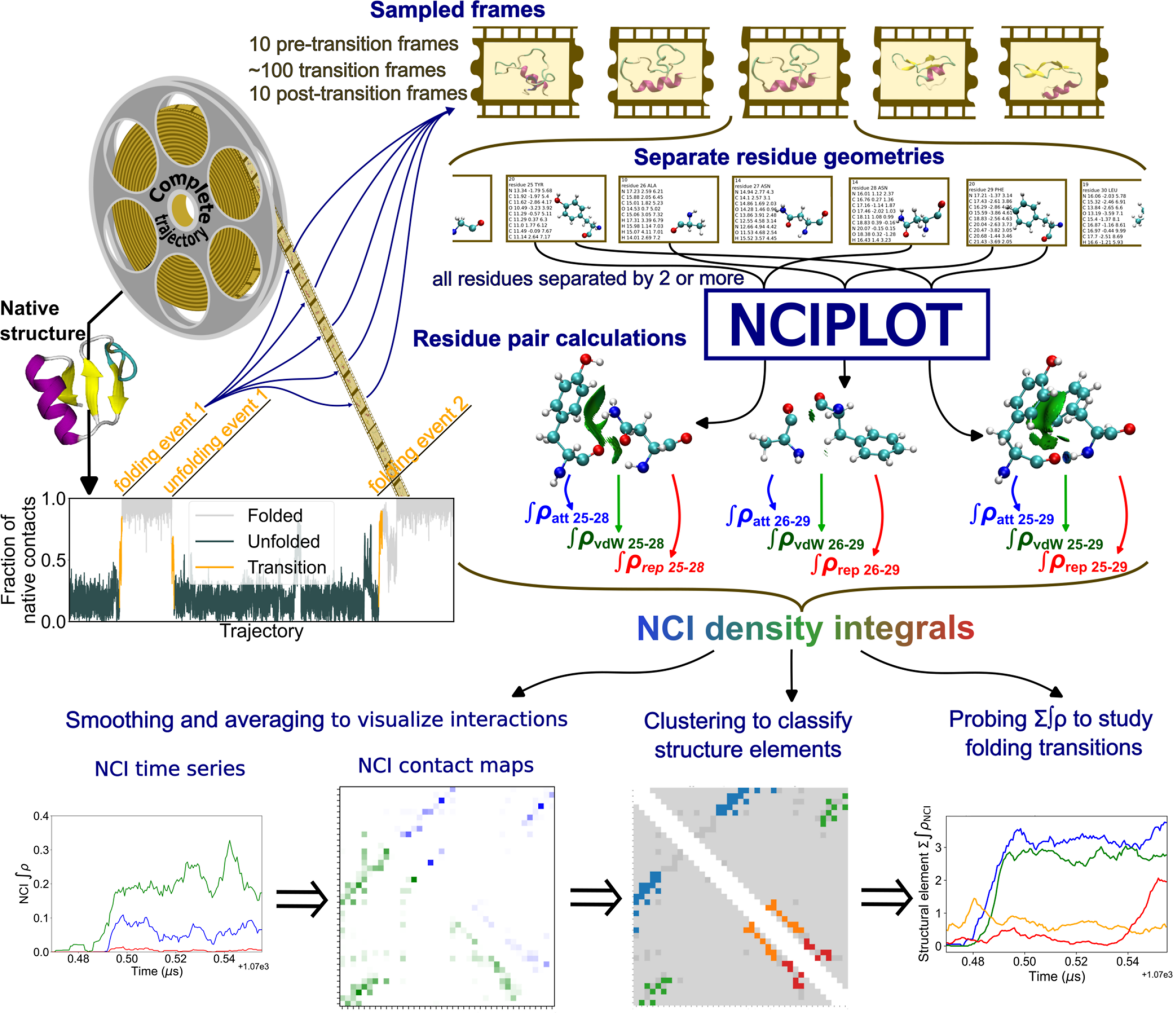
Proposed workflow for the estimation of NCI density integrals from MD simulation data.

### Workflow for the Analysis of Density Integrals from MD Simulation Data

We start from an MD data set that may contain rare events like protein folding or ligand binding. These data sets are often considerably large, and hence we will typically only analyze a subset of the data. Frames from the simulation data sets can be selected in an unbiased way (i.e., by dumping equally spaced or randomly chosen snapshots from the simulation trajectories) or paying particular attention to rare events identified using a progress coordinate. In line with the work by Shaw et al.,^23,24^ here we used the fraction of native contacts (*Q*)^25^ to select frames from the native, unfolded and transition state ensembles, to be analyzed separately. Also, we are interested in mechanistic information from NCIs during folding and unfolding events. To identify transition paths that exclude recrossings, we used transition-based assignment^26^ between cutoff values of *Q* for bound and unbound states (0.9 and 0.1, respectively, see Figure S1). A sampling step was determined to dump approximately 100 evenly spaced frames for each characterized transition. Furthermore, 10 equally spaced frames from the trajectory immediately before and after these transitions were included to adequately define the boundaries.

For each simulation snapshot, the coordinates of each individual amino acid residue are written in separate files (see Figure 1). Then, all pairwise interactions between residues separated by at least three positions in the sequence are calculated using these coordinates. The treatment of residues as isolated fragments is compatible with the promolecular approach used for the estimation of electronic densities (see below). This approach enables a computationally efficient yet robust representation of the NCIs of the system, and can be used to provide detailed integrals for all relevant residue pairs and to classify them by interaction type and by partners involved. Subsequently, the electronic density integrals corresponding to the interactions between the residue pairs are compiled and processed using a series of Python scripts.

### Density Integral Calculations

Density integrals were calculated using the NCIPLOT4 software package using promolecular densities.^18^ These densities are approximations of the true electronic density obtained as the sum of predefined densities for each isolated atom.^27^ Although these molecular fragments include backbone atoms with incomplete valence shells, this does not affect the promolecular densities because the atomic contributions are predefined and are not altered by the missing bonding partners. Moreover, since interactions between amino acid residues linked by a polypeptide bond are not considered, we avoid introducing artifacts into the analysis caused by calculating the interactions between bonded atoms as if they were part of two separate molecular entities.

These calculations generate three-dimensional grids enveloping the studied system in which the electron density (ρ), the reduced density gradient (*s*) and the eigenvalues of the Hessian matrix of the electron density (λ_*n*_) are evaluated.

The regions between two molecular fragments, characterized by low values of ρ and *s*, and significant density contributions from each fragment are associated with NCIs between these fragments.^20,28^ The Hessian eigenvalues in these regions indicates how the electronic density maximally varies in three orthogonal directions. The largest among these values (λ_1_ ≤ λ_2_ ≤ λ_3_) corresponds to the pronounced density minima found along the path between the two fragments, while λ_1_ and λ_2_ correspond to the eigenvectors in the plane normal to it. Similar to covalent bonds, bonding interactions exhibit electron density enrichment in the normal plane, resulting in λ_1_ ≤ λ_2_ < 0. In contrast, a positive eigenvalue shows the density being depleted in one or more perpendicular directions, signaling a repulsive character. Dispersion or van der Waals interactions, being more diffuse, typically yield eigenvalues close to zero and are therefore less straightforward to interpret, although they can be recognized by their generally low electron density accumulation.

In NCIs, λ_3_ is dominated by the interfragment separation and provides little information on the nature of the interaction, while λ_1_ is generally more negative and strongly correlated with λ_2_, adding no additional interpretative value. Hence, in NCIPLOT interaction regions are classified according to the sign and magnitude of sign­(λ_2_)­ρ compared to certain thresholds. For this work, we employed the empirically validated and widely utilized thresholds of −0.02 and 0.02 to differentiate regions as either strongly attractive, weakly attractive (typically associated with vdW forces) or repulsive. The densities of the interaction regions can be integrated, yielding quantitative results. The density integrals for the transitions were smoothed using a moving average with a window size of 10 sampled frames. Further methodological details, along with illustrative examples demonstrating the decomposition of interactions by residue and the analysis of intramolecular contacts, are provided in the Supporting Information (see Figures S2–S4).

### MD Data Sets

We apply our workflow to two well-characterized ultralong protein folding trajectories produced by the group of Shaw.^23,24^ These trajectories capture multiple transitions at atomic resolution and can offer detailed insight into the folding reaction. Specifically, we analyze 4 replicas of the K12M mutant of NTL9 (39 residues) with lengths of 1052, 990, 389, and 377 μs, produced using the CHARMM22* force field,^29^ and one trajectory of FiP35, a mutant of the Pin1 WW domain (35 residues) with a length of 100 μs using a modified version of the AMBER99SB force field.^30^ The NTL9 trajectories include a total of 18 folding and 14 unfolding events, whereas the FiP35 trajectory includes 4 folding and 3 unfolding events. Hence, these trajectories capture multiple transitions at atomic resolution and can offer detailed insight into the folding reaction.

## Results and Discussion

### NCIs in Protein Folding Trajectories

In this analysis, interactions among residue pairs are captured as inter-residue density integrals, encompassing the combined electronic density arising from spatial regions with meaningful contributions from pairs of amino acid residues. These densities can convey actual molecular interactions, taking into account orientation, interacting atoms, as well as the influence of neighboring groups. Additionally, this approach allows for the quantitative separation of the contributions arising from different interaction types, namely, attractive, vdW forces, and repulsive, between two molecular fragments. This decomposition provides a rich view of the interactions driving the protein structure and dynamics.

In Figure 2, we present an example of how NCI densities can give a more granular depiction of the interaction between different entities. As observed in the four example frames, NCIs arise from one, or more commonly, multiple basins between the interacting residues, meaning closed spatial regions where the electron density exhibits features characteristic of NCIs. These basins are classified based on their sign­(λ_2_)­ρ values, yielding the three plotted density integrals. As it would be expected, shorter distances generally correlate with larger NCI integrals, as closer contact of the residues allows for a larger overlap of the electronic density of the two residues. Basins with sign­(λ_2_)­ρ values between −0.02 and 0.02 are shown in green, and correspond to broad surfaces typically assigned to vdW forces. Although vdW interactions depend on the chemical nature and orientation of the interacting moieties, they are nearly ubiquitous, contributing significantly to the overall protein stability. Basins with sign­(λ_2_)­ρ values below −0.02 are attributed to attractive interactions, and those above 0.02 are assigned to repulsive contacts. Attractive interactions, depicted as blue surfaces, indicate locally enriched electron density and are predominantly associated with hydrogen bonds, emerging as small disks between the acceptor and donor groups. Unlike vdW forces, hydrogen bonds require specific interacting groups and precise directional alignment, making them less readily formed despite often being stronger. Lastly, repulsive regions are shown as red surfaces, representing areas of relatively elevated but locally depleted electron density. These regions are rare and typically have minimal impact, as proteins naturally adopt configurations that minimize destabilizing effects.^31^

**Figure 2:**
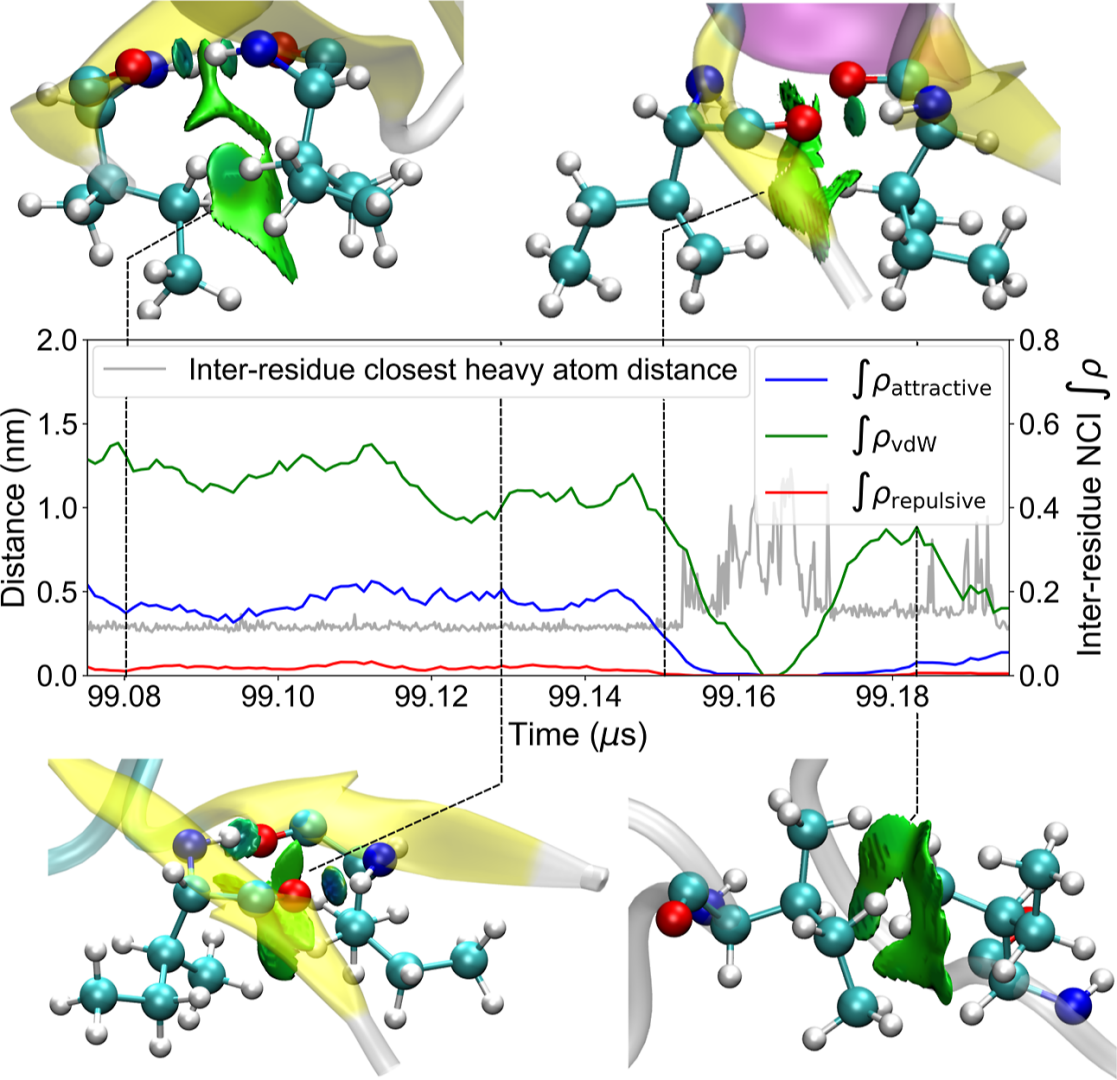
Time series data of pairwise distances between Ile4 and Ile37 and corresponding inter-residue densities for attractive, repulsive and vdW interactions during an unfolding event of protein NTL9. A selection of snapshots is included, showing all the atoms of these residues, a cartoon representation of secondary structure and inter-residue NCI isosurfaces.

The two interacting Ile residues shown in Figure 2 belong to opposing fragments of an antiparallel β-sheet. In the first frame, two distinct interaction types are formed between these residues. Hydrogen bonds, which contribute to the attractive interaction integral, are represented by the two blue discs between backbone hydrogen donors and acceptors. Below the discs, we show a broad green surface arising from the proximity of other atoms. These surfaces represent vdW interactions, appearing primarily between proximal side chains that can form stabilizing dispersion interactions. In the second frame, there are similar inter-residue interactions, however the NCI integrals show some noticeable differences. The vdW surface and its corresponding density integral have decreased as the longer part of the side chains rotate away from the interaction region, reducing the electron density overlap between these residues. Conversely, the attractive integral has risen, as one of the hydrogen bonds shortens, going from 2 to 1.8 Å. In the third frame, the β-sheet has begun fraying, as the protein approaches its unfolded state, with the changes in integrals and distance indicating this process. Both residue side chains drift further apart, and one of the hydrogen bonds is broken. In the final frame, the contact is no longer part of the secondary structure element, and no backbone hydrogen bonds are present. Here, contacts occur solely between the apolar side chains, resulting in a larger pairwise distance and an effectively vdW-only interaction. Therefore, while the unfolding process is captured by both the distance and the NCI density integral, the latter offers considerably more information. It bears noting that the correlation of NCI data to individual geometries is affected by the use of a smoothing window on the density integrals.

### Contribution of Hydrogen Bonds to NCI Integrals

Hydrogen bonds play a central role in stabilizing protein structures and are a defining feature of the interactions between both small groups and larger structural elements.^32^ These interactions are not only abundant in biological macromolecules but also relatively strong among NCIs.^33^ However, their formation requires specific geometric alignment between donor and acceptor, imposing considerable conformational constraints on the participating residues. This structural specificity leads to recurring patterns in protein conformations, making hydrogen bonds highly informative about the overall structure and stability of proteins. In the context of NCI analysis, hydrogen bonds manifest as regions of attractive interaction density, typically visualized as blue lentil-shaped isosurfaces located between donor and acceptor atoms.^33^ Attractive NCI density for biomolecules, such as the studied proteins, consist mostly of these hydrogen bonds, with larger density integral reflecting stronger bonding.

In Figure 3A we present the correlation between the cumulative inter-residue attractive density and the number of hydrogen bonds detected with the Baker–Hubbard model^34^ for a series of sampled NTL9 conformations. We find a strong correspondence between attractive NCI regions and the hydrogen bonds captured through geometry-based tools. The correlation between the number of hydrogen bonds and attractive density is evident, resulting in a value of *R* ^2^ = 0.62 for all the data. The observed spread in the data can be mainly attributed to two factors. First, and most relevant, hydrogen bonds vary in strength across protein conformations due to differences in geometry, namely, distance and angular alignment, which impacts their contribution to the attractive density despite being equally counted by the Baker–Hubbard model. Second, predominantly vdW interaction regions may include minor attractive contributions as the electronic density surpasses the empirically defined threshold in some points, adding further variability.

**Figure 3:**
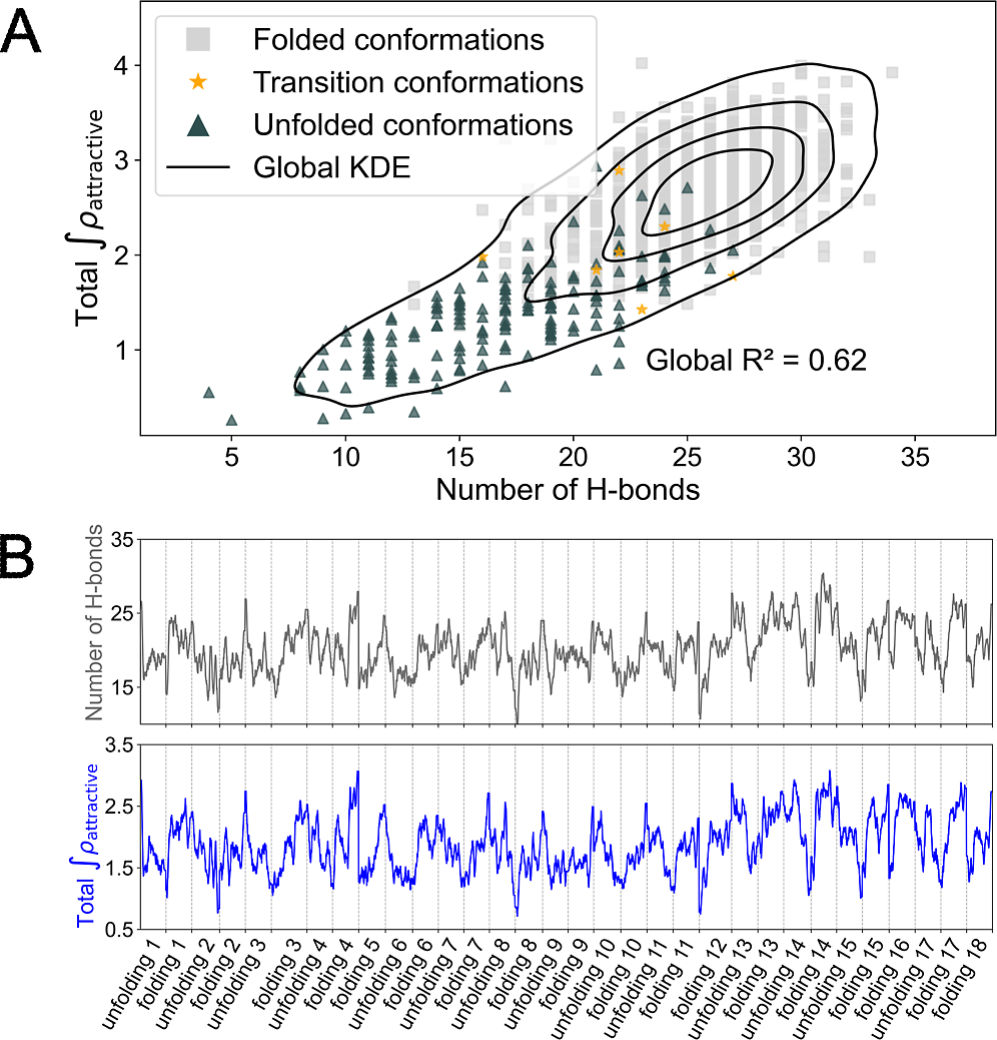
(A) Scatter plot of hydrogen bonds (Baker–Hubbard model) versus the total ∫ρ_attractive_ for folded, midtransition and unfolded NTL9 conformations. The number of data points in each ensemble is proportional to the population in the complete data set. (B) Number of hydrogen bonds detected and total ∫ρ_attractive_ (blue) along every NTL9 folding or unfolding event, a smoothing window of 10 data points was used for both quantities.

If we now focus in the distribution of hydrogen bonds in the different conformational states of the protein, we find that unfolded conformations generally show fewer inter-residue hydrogen bonds, while folded ones tend to show the most, which is reflected in the attractive densities of these conformations (see Figure 3A). These inter-residue attractive interactions can be primarily ascribed to backbone hydrogen bonds from which the secondary structure arises. Folded states, in turn, display a denser network of such stabilizing contacts and correspondingly higher total attractive densities. This aligns with the idea of hydrophobic collapse, where the native state maximizes internal stabilizing contacts while the unfolded state presents fewer internal interactions, but compensates this with increased protein–solvent interactions.

We have also investigated whether the contribution of hydrogen bonds also tracks the increase of attractive interaction density across folding events. In Figure 3B, we show the smoothed progression of both hydrogen bond count and the summation of attractive density across all sampled transitions for NTL9. Both quantities follow similar trends, rising during folding processes and declining during unfolding events, leading to comparable time-course profiles. Taken together, these results suggest that identifying residue pairs involved in significant attractive interactions may provide insights into the formation and stability of hydrogen bonds stabilizing secondary structure elements.

### NCI Contact Maps

The calculation of the NCI density integrals between every residue pair in a protein enables the depiction of its interaction network as a contact map, a widely used tool for the study of protein interactions, though traditionally based on interatomic distances.^35^ In Figure 4, we compare the NCI densities between all NTL9 residue pairs separated by at least three positions in the protein sequence with the distance-based contact map (in this case, calculated using inverse interatomic distances). We present separate NCI contact maps for the native, unfolded and transition state ensembles, which are defined based on the fraction of native contacts (see Materials and Methods), whose distribution for each of the ensembles is shown in Figure 4A. We note that in the case of the unfolded ensemble, the probability distribution of *Q* exhibits a long tail extending toward considerably large values, which likely reflects native-like interactions preexisting in the unfolded ensemble or structuring during recrossings.^25,36^

**Figure 4:**
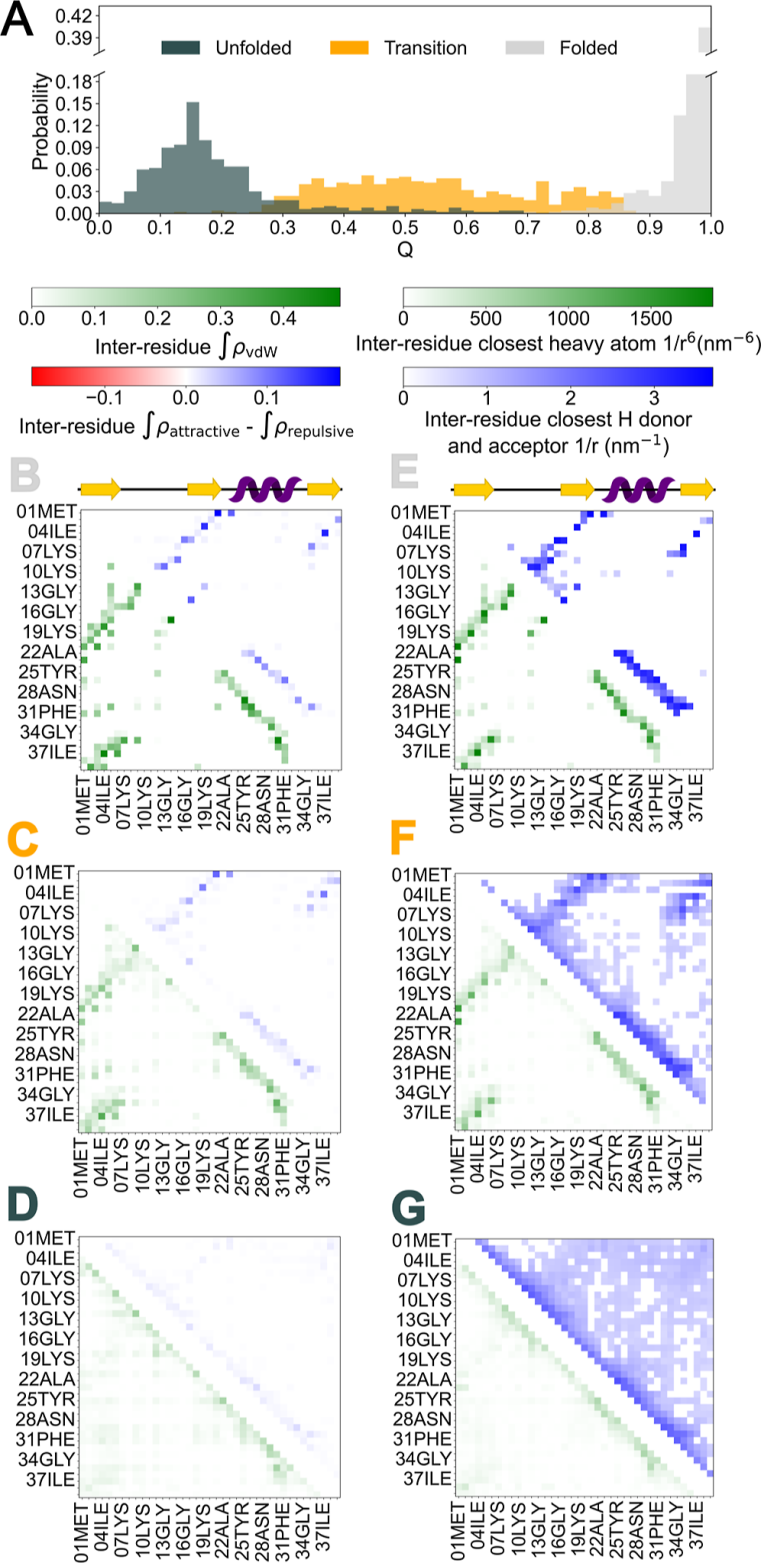
(A) Distribution of the fraction of native contacts for the folded, unfolded and transition ensembles. (B–D) Integrated NCI densities for vdW interactions (lower triangle) and attractive minus repulsive interactions (upper triangle) for the folded, transition and unfolded ensembles, respectively. (E–G) Same for the inverse distances among the closest heavy atoms of each residue pair to the 6-th power (upper triangle) and inverse distances for the closest atoms in hydrogen bonds for each residue pair (lower triangle). The positions of β-sheets (yellow) and α-helix (purple) in the native state are shown above panels B and E. In all cases, only pairs with a sequence separation larger than 3 residues have been considered. Results were averaged over 500 randomly selected snapshots in each ensemble.

The inter-residue NCI density integral maps for the folded, transition, and unfolded ensembles (see, respectively, Figure 4B–D) indicate a considerable loss in intensity of NCI as the protein loses its native structure. As expected, NCI integrals both for the vdW and attractive interactions are large in the folded ensemble, and die out as we move into the unfolded state. These NCI contact maps bear significant similarity with the corresponding distance contact maps (see respectively, Figure 4E–G), here calculated using the inverse distances for the hydrogen bonds and the inverse distances to the 6-th power for the heavy atom contacts (see Figure S5 for the comparison to just inverse distances). This mapping is due to the inherent relationship between inverse distances and interaction strength of NCIs of electrostatic and vdW forces. Both types of maps reveal recurring patterns corresponding to secondary structural elements. Diagonals ascending from left to right represent interactions between antiparallel β-sheets, while diagonals positioned near the bisectrix, ascending right to left, correspond to α-helical contacts between residues approximately four positions apart. In these maps, one α-helix and two β-sheets are observable: one formed between the first and second strands, and the other between the first and third strands in the sequence. However, key differences arise due to the sensitivity of NCI densities to more than just proximity. Factors such as the number of interacting atoms, their precise spatial arrangement, and the chemical nature of the interacting groups simultaneously contribute to the interaction strength. As a result, NCI and distance contact maps differ in significant ways, revealing the additional structural and chemical detail captured by this method (see below).

The folded ensemble maps present clear and well-defined maps, reflecting primarily the structural elements characteristic of the native state. The transition ensembles show the same structural elements, albeit with reduced intensity, consistent with transient partial folding. Whereas the unfolded map presents significantly more dispersed interactions. Interestingly, several residue pairs near strong interactions in the distance maps show much weaker signals in the NCI maps. This can be attributed to spatial proximity effects: residues close to a strongly stabilizing contact may appear nearby in space yet interact only weakly with each other. While these “bystander” pairs can register strongly in distance maps, they contribute only faintly to the NCI density maps. This contrast highlights the advantage of NCI maps in discerning which interactions are truly relevant for structural stabilization.

Another important point of divergence in the maps arises between the attractive NCI densities and inverse hydrogen bond distances. The hydrogen bond distances are computed by identifying every recorded hydrogen bond, even those formed in just a single frame, and tracking the donor–acceptor distances across all frames. The shortest distance is used for each residue pair and frame to compute the inverse. As a result, some residue pairs with low average NCI density may still show high inverse distances, reflecting geometries that are close enough to be noted but poorly aligned for hydrogen bonding. This demonstrates the value of using a unique variable, for instance, the NCI density, to properly capture more complex interactions, such as hydrogen bonds.

### Protein Folding Mechanisms from NCIs

NCIs may also be useful to extract important information regarding the mechanisms of protein conformational transitions. In the case of the protein folding trajectories, we focus exclusively on the reactive segments where the protein traverses between the folded and unfolded states (i.e., the transition paths). Specifically, we interrogate whether structural motifs characteristic of a protein’s architecture that concertedly form and break can be derived from the sets of pairwise NCIs. We do this using time-series clustering of residue pairs based on their NCI densities for folding and unfolding events of NTL9 (see details in Supporting Information Methods). In Figure 5A, we show the result of this analysis, where the grouping captures the secondary structural elements in the native structure of the protein. The β12 motif (blue) contains the interactions between the β-strands spanning Met1–Phe5 and Glu17–Val21. The β13 motif (green) involves the interactions between the same initial strand and a third strand covering Leu35–Ala39. The α1 motif (orange) consists of a series of contacts between residues Ala22 to Phe31 that give rise to the first segment of the α-helix. The tail of the α-helix comprising contacts between residues Ala28 to Gln33, and a set of interactions between the last helix turn and the adjacent coil up to Ala36, make up α2 (red).

**Figure 5:**
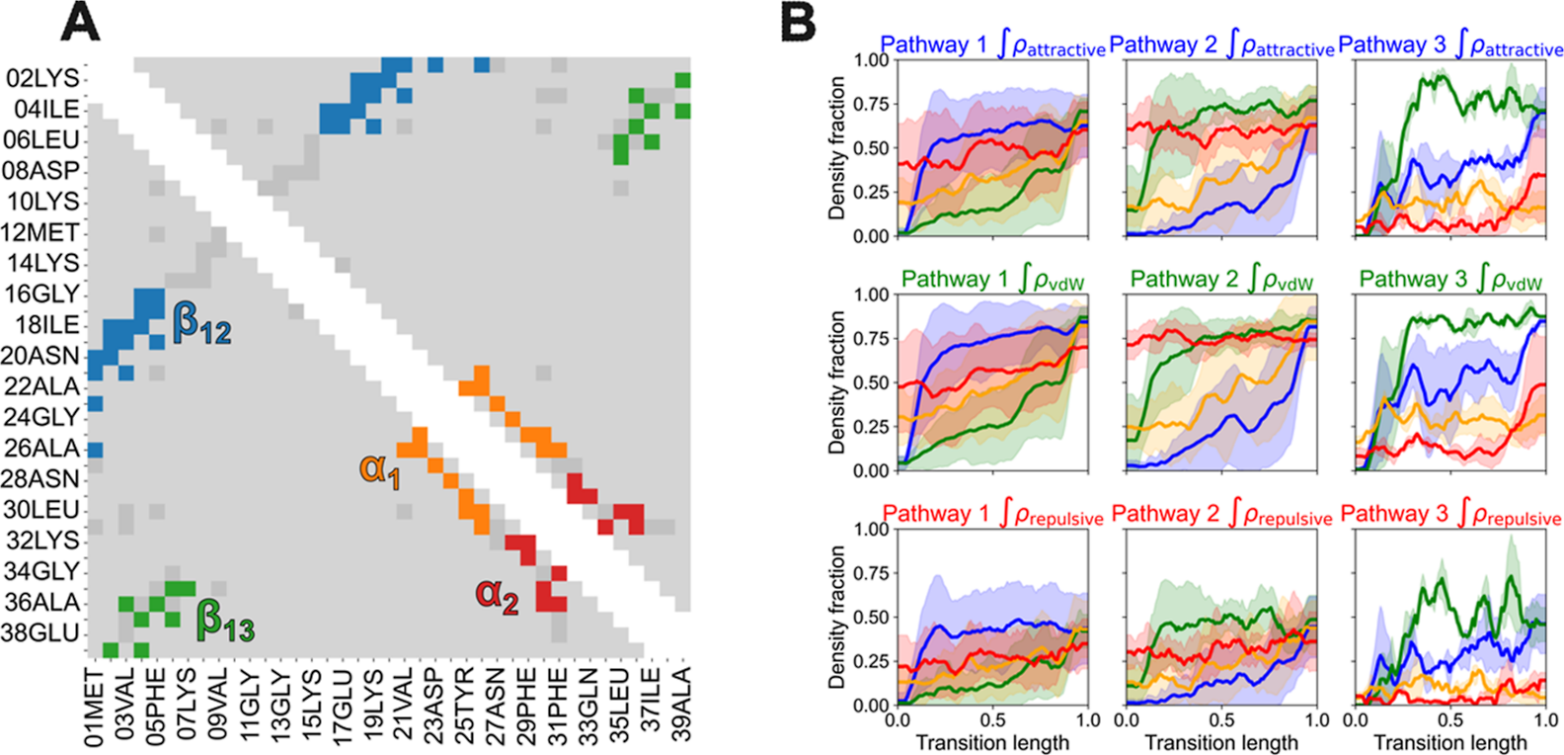
(A) Residue pair clustering for NTL9 based on the 3 separate NCI density integrals along the 32 determined transitions. (B) Pathways emerging from clustering, including the average and standard deviation of the normalized density of each characterized structural element in each pathway. Pathways 1, 2, and 3 represent 56%, 38%, and 6% of the characterized transitions, respectively.

Outside the interpretable groupings, we find many other residue pairs, shown in dark gray in Figure 5A. These include the turn spanning Lys7–Lys15, which stacks residues similarly to a β-sheet. Another example of interactions in this cluster are those between the first β-strand—namely Met1, Val3 and Phe5—and the α-helix, specifically Leu30 and Phe31. While these contacts are certainly relevant to the protein conformation, they cannot be ascribed easily to a specific process during folding or unfolding. For simplicity, we leave them out in the subsequent analyses. Finally, most residue pairs (light gray) largely fail to show coordinated changes across transitions and are treated as noise.

Having used the NCI analysis to group residue pairs in clusters, we can analyze whether different mechanisms—defined as events with a different order of motif formation, as before^29^—are identifiable. Multiple metrics point to three clusters yielding the best separation among clusters (see Supporting Information text and Figures S6–S10 for further details). In Figure 5B we show the NCI density fractions for attractive, vdW and repulsive interactions for folding events and (reversed) unfolding events corresponding to these different folding pathways. The first one, representing 56% of the transitions, starts with intermediate density fractions for α1 and α2, while contacts corresponding to β12 and β13 are entirely missing. This indicates substantial α-helix folding at the start of the transition, while the β-sheets are completely unfolded. In the early stages, the density fraction of β12 increases sharply, indicating that this structural motif forms first in the transition paths, while the densities of the other three motifs increase more gradually. Throughout the remainder of the transition, β12 density remains stable, α1, α2 and β13 continue to rise and eventually reach their final value. During most of the transition, α2 exhibits higher density than α1, and β13 presents the lowest density among the motifs. Therefore, these transitions, although starting with the α-helix partly formed, involve first the formation of β12, followed by the completion of the α-helix, and finally the formation of β13. The second pathway, present in 38% of the transitions, again starts with a moderately high density fraction for α2, but the order of β-strand folding is reversed relative to the pathway 1. Early in the transition, β13 forms rapidly, followed by α1 and β12. β12 comes last in this pathway, increasing sharply only at the end. Finally, the third pathway represents only 6% of the transitions. It starts with all four motifs presenting minor relative densities. The first element to fold in these two transitions is β13, which is subsequently followed by β12, α1 and finally by α2.

The two predominant pathways characterized with our methodology are consistent with those reported by Lindorff-Larsen et al.^24^ and also with a more recent investigation using Markov state models.^37^ Specifically, Lindorff-Larsen et al. characterized folding mechanisms based on the point in the transition at which each residue adopts a native-like structure.^24^ The first one starts with the formation of the β12 sheet, followed by the formation of the α-helix with the region 28Asn–30Leu being the last to adopt this conformation. Lastly the β13 sheet is formed. The second pathway starts with the residues 31Phe–35Leu adopting its native-like conformation, and then the formation of the β13 sheet. After that, the α-helix is completely formed, and last, the β12 sheet is formed.

While *Q* has been proven to be a robust collective coordinate for protein folding,^25,38^ notable discrepancies may exist between the conformational states at the boundaries of transitions, reflecting the inherent diversity of folding pathways and the structural heterogeneity of the unfolded and folded ensembles. In some of the transitions, we find that the NCIs for some of the structural elements are already preformed in the unfolded structure and some others may not be completely formed in the folded one. These discrepancies among transitions add to the heterogeneity of our data, further contrasting the behavior of distinct structural features. Hence, this analysis is able to distinguish the many less important residue contacts to those determining the protein structure and also separate the latter among different structural elements. Importantly, the classification of residue pairs was performed exclusively using NCI data, without incorporating sequence information or spatial context. This demonstrates that interaction densities alone are sufficient to identify structural features and differentiate their roles across folding pathways.

### Protein–Solvent Interactions along Protein Folding

Water molecules are extremely important in conformational transitions like protein folding,^39^ as desolvation mediates the collapse of the protein core.^40^ The role of water in biomolecular transitions can be further understood from the analysis of NCIs. Here we focus on a different trajectory, that of the FiP35 WW domain,^23,41^ a smaller protein with two β-hairpins. Here, we analyze the interactions of all pairwise residues and between each residue and the surrounding water molecules along a representative folding event (see Figure 6A). Upon folding of FiP35, there is a concomitant increase in the value of *Q* and a decrease in the accessible surface area of the residues of the protein that form the β-strands (see Figure 6B,C). We are interested in the difference between NCIs in the folded and unfolded states, i.e. Δ∫ρ for different interaction types. Positive (negative) values of Δ∫ρ are indicative of gain (loss) of density for the corresponding interaction upon folding. We find that these changes for both the intraprotein and solvent–protein interactions are able to track the folding event (see Figure 6D,E).

**Figure 6:**
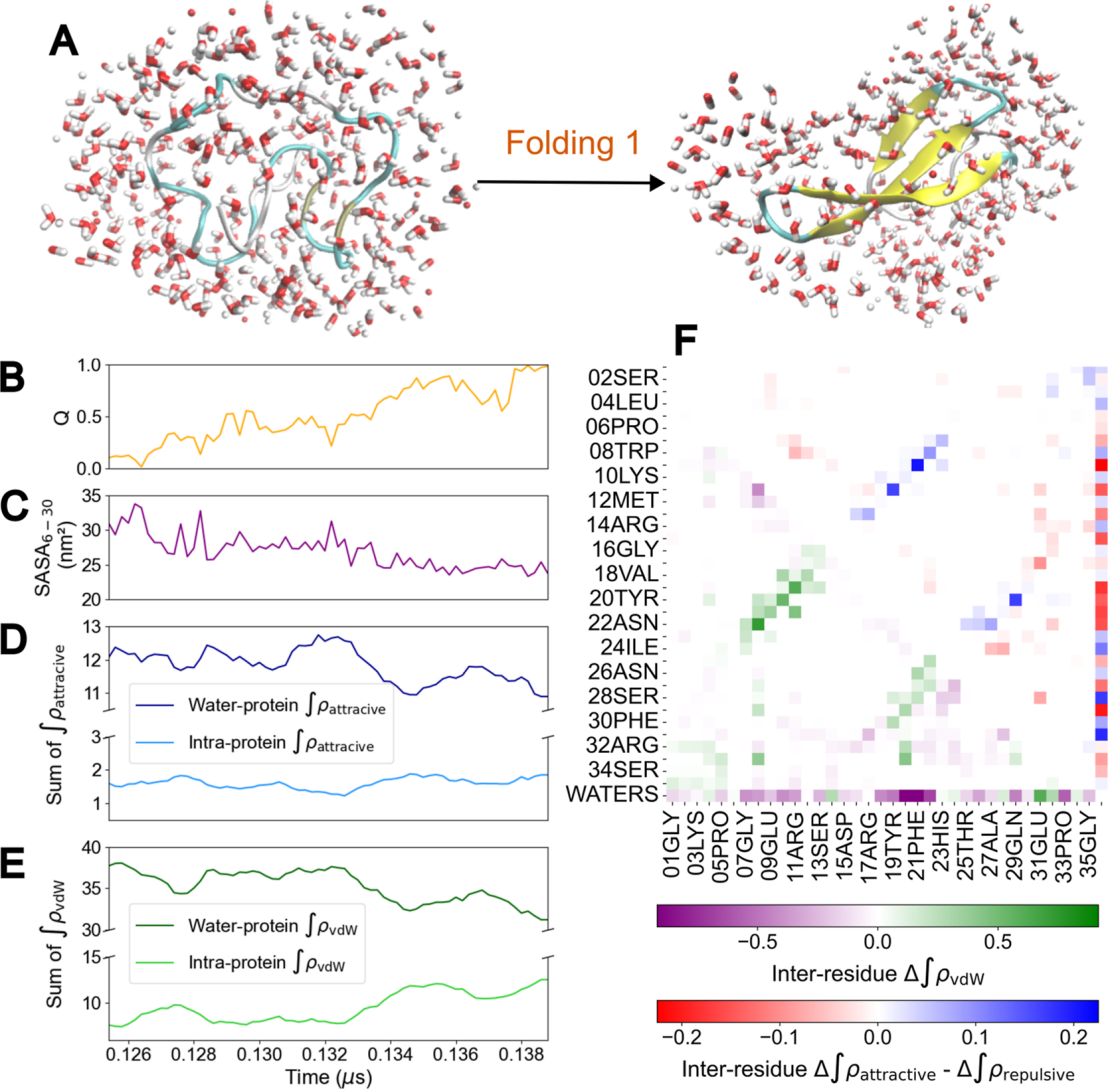
(A) Representation of FiP35 and the water molecules surrounding it at the start and end of a folding event. (B) Native contact fraction throughout the transition. (C) Solvent-accessible surface area (SASA) for residues 6–30, (tails are excluded to focus on the protein core that forms the β-hairpins). (D) Sum of the attractive density integrals between each residue and surrounding water, as well as between all residue pairs. (E) Sum of the vdW density integrals between each residue and surrounding water, as well as between all residue pairs. (F) Difference in NCI density integrals between the end and start of the folding event.

In Figure 6F we show the NCI contact map, here showing differences in NCI integrals between the end states of the folding transition. The two antiparallel β-sheets corresponding to the hairpins stand out as having an increase in both attractive and vdW interactions. Additionally, the analysis reveals changes in how water molecules interact with different regions of the protein throughout the transition. The first two β-strands, spanning Gly7–Ser13 and Arg17–His23, present notably positive density differences in both vdW and net attractive-minus-repulsive NCI densities in their mutual interactions, while showing primarily negative density differences in their interactions with the waters. These regions transitions from a solvent-exposed coil in the unfolded state to a more buried configuration as pair of a folded hairpin. This shift enhances the interactions between them, while reducing their solvent accessibility. The third strand (Ala27–Phe30) shows mixed results, indicating that some residues in this region maintain or even increase their interactions with water, while others become more buried.

## Conclusions

In this work, we developed an NCI-based approach for examining protein interactions and applied it to the study of folding pathways. This tool enables a detailed dissection of the interactions between different protein fragments by computing three integrals per residue pair, corresponding to attractive, vdW, and repulsive density regions. By offering both qualitative and quantitative insights, this technique provides a granular view of NCIs in large biomolecular systems while maintaining a modest computational cost.

The NCI integrals not only capture contacts among amino acids, but offer quantitative insight into the strength of these interactions. Attractive densities primarily reflect hydrogen bonding, which plays a central role in the formation and stability of protein secondary structures. vdW densities account for a broad range of interactions, such as dispersion forces and π–π stacking, that occur throughout the protein. These interactions are present in almost all residue contacts and typically constitute the largest share to the total integral density. As such, vdW integrals capture both interactions directly involved in structural element formation and those that stabilize the protein more generally. While repulsive densities are consistently minor contributions—due to proteins favoring conformations that minimize these forces, they can still be meaningful in cases where steric hindrance or close contact impacts folding or packing.

Beyond internal residue–residue interactions, this framework also accommodates interactions beyond the protein itself, such as those with solvent or ligands. By computing water–residue NCI integrals, it becomes possible to assess solvent exposure and the stabilizing or destabilizing nature of hydration across the protein. This offers a more complete depiction of protein dynamics and stability, especially in cases where solvent interactions significantly influence protein stability or function.

Applying this approach to multiple folding trajectories, we characterized distinct groups of residue contacts that form the major structural elements of NTL9. These results were also used to delineate the folding and unfolding pathways, revealing distinct transition routes that agree with prior studies. The approach quantitatively tracks how interaction strengths evolve over time, providing a physically grounded view of conformational change. Beyond the application presented in this work, this analysis could be used to compare folding mechanisms under different conditions, such as temperature, salt concentration, or pH. For example, Chung et al. observed substantial changes in both the transition and folded ensembles under varying conditions, and the approach described here could provide a basis for understanding these differences.^42^

It is worth noting that the current promolecular framework does not differentiate between orbital contributions from charged molecular fragments. This limitation could be addressed through orbital libraries that account for the chemical environment of each atom, such as the approach proposed by Genoni et al.,^43^ without imposing a significant computational burden. Also, although here we have focused on the case of protein folding from classical MD trajectories, the approach is general and can be applied to other types of simulation data, including ab initio MD, QM/MM calculations, or molecular dynamics simulations guided by experimental restraints, such as those derived from NMR or cryo-EM data.^44^ Having access to more accurate or higher-level calculations is particularly interesting, as it can ensure a more faithful representation of the interactions in the system, and therefore a more reliable analysis.

Given its accuracy, interpretability, and computational efficiency, this NCI-based framework holds promise for broader applications in protein science. It provides a robust foundation for future studies of protein dynamics, allosteric regulation, and ligand interactions. As the methodology continues to evolve, particularly with improved representations of charged residues and the possibility to establish a quantitative connection with interaction energies,^21^ it is well-suited to become a valuable complement to existing structural and dynamical analysis tools in the study of complex biomolecular systems.

## Supplementary Material



## Data Availability

The analyses presented in this work were carried out on the trajectories made available by D. E. Shaw Research. The scripts developed for this work as well as a tutorial for their utilization are publicly available at https://github.com/BioKT/NCIfolding.

## Notes

Further information on the NCIPLOT calculation and clustering methodology, as well as the figures mentioned in the text (PDF)
