# Supplementary material for: Resolving Molecular Interactions in Protein Folding Trajectories with NCIPLOT
Source: J Chem Inf Model. 2025 Sep 16;65(19):10613–23. doi: 10.1021/acs.jcim.5c01501 (PMC12529773; doi:10.1021/acs.jcim.5c01501)
Supplement: Supplementary file 1 [file ci5c01501_si_001.pdf]

Supporting Information: Resolving molecular interactions in protein folding trajectories with NCIPLOT

Asier Urriolabeitia,* Julia Contreras-García, David De Sancho, and Xabier López*

Polimero eta Material Aurreratuak: Fisika, Kimika eta Teknologia, Kimika Fakultatea, UPV/EHU & Donostia International Physics Center (DIPC), PK 1072, 20018 Donostia-San Sebastian, Euskadi, Spain

E-mail: asier.urriolabeitia@ehu.eus; xabier.lopez@ehu.eus

Trajectory analysis and geometry extraction for NCIPLOT4 calculations were carried out using the all-atom NTL9 and Fip35 simulations performed and made publicly available by Shaw's group.

In this work, native contacts for Q-value calculations were derived from structures predicted by AlphaFold2. In contrast, Shaw et al. defined native contacts based on the central structure of the most populated cluster, obtained by clustering 50,000 simulation snapshots using Cα-RMSD. Despite this difference in methodology, we observed strong agreement between the native contact fractions calculated using our simplified approach and the original clustering-based method. Figure S1 shows the native contacts fraction for the complete D. E. Shaw et al. trajectories.

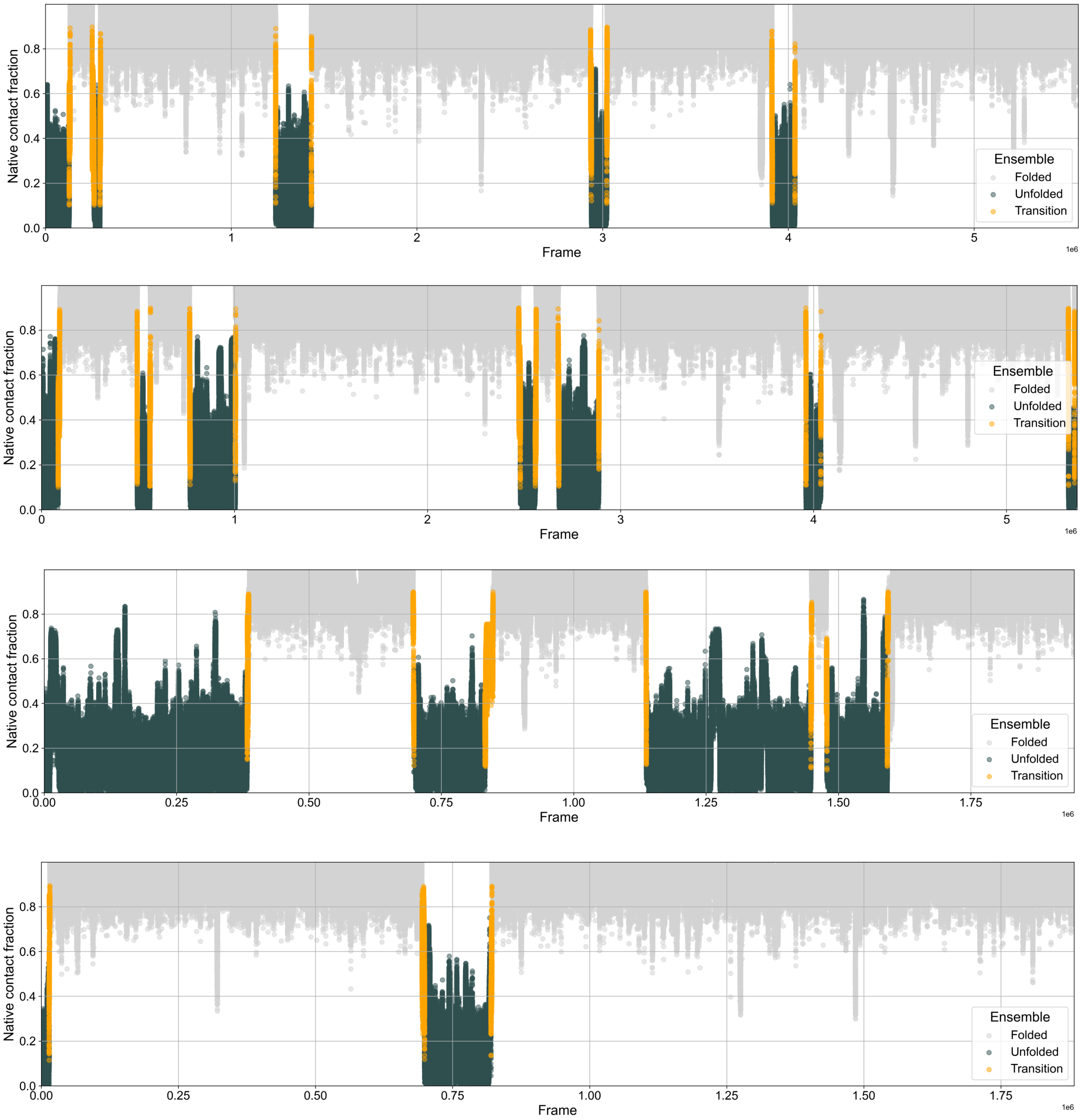

Figure S1: Native contact fraction for the four NTL9 trajectory replicas carried out by D.E. Shaw et al.

NCIPLOT4 calculations were performed using the following keywords:

2

RESIDUE1.xyz

RESIDUE2.xyz

COARSE

INCREMENTS 0.1 0.1 0.1

INTERMOLECULAR

INTERCUT 0.85 0.75

INTEGRATE

OUTPUT 1

RANGE 3

-0.2 -0.02

-0.02 0.02

0.02 0.2

In these calculations, promolecular densities are computed using promolecular orbitals at the atomic xyz positions, evaluated on a grid of cubes with 0.1 Å in each direction. Densities are limited to regions where both fragments contribute electronic density, with a maximum contribution of 85% allowed from each fragment. The resulting densities are integrated and partitioned in the three defined ρ ranges. The resulting $\int \rho^n_{\text{range}}$ from this calculations are outputted in a .out file, summarizing the numerical results from the calculation. The n=1 integrals from each range are parsed with a python script to be processed by our analysis pipeline.

It bears noting that $\int \rho^n_{\text{range}}$ for other n values are also available and their utilization could be easily be implemented in the pipeline for the presented analyses or other purposes. For simplicity and interpretability this work is limited to the analysis of $\int \rho^1$

The densities of interactions between large structural features are perfectly captured by the interactions between pairs of individual residues making them up. Figures S2 and S3

examplify it presenting the densities between the 01Met-05Phe and 17Glu-21Val strands mostly corresponding to the densities in $\beta 12$ and those between 02Lys-06Leu and 35Leu-39Ala strands, capturing $\beta 13$.

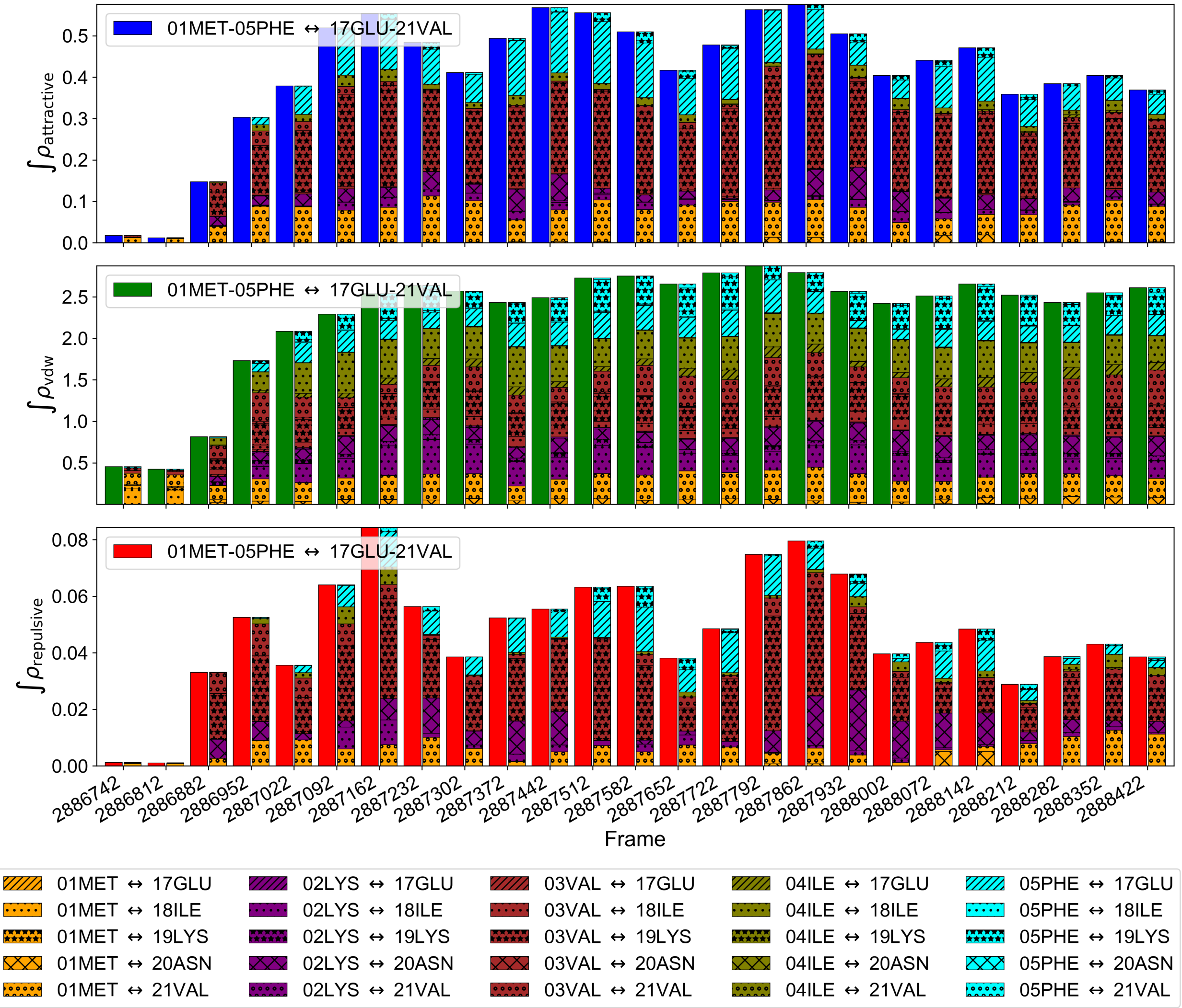

Figure S2: NCI densities between the first and second β-strand and those between all possible combinations of the individual residues in said strands

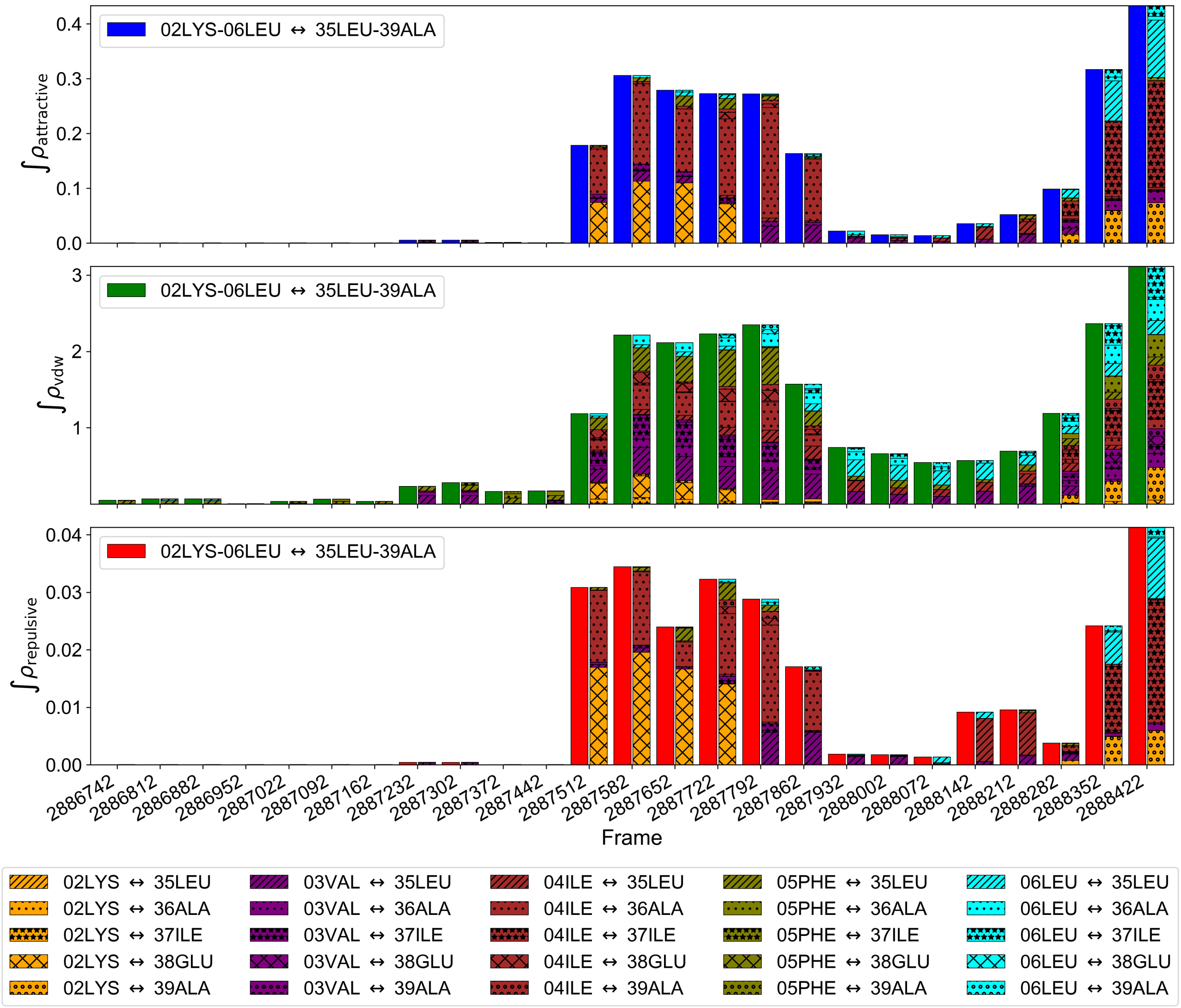

Figure S3: NCI densities between the first and second β-strand and those between all possible combinations of the individual residues in said strands

This shows our approach perfectly captures interactions in structural motifs composed by two groups of residues separated in the protein sequence, namely β-sheets or tertiary structure interactions. This comparison is not possible for structural elements composed by a sequential group of residues, namely α-helices. In said cases, where only one molecular fragment is inputed, INTERMOLECULAR keyword of NCIPLOT is not applicable, resulting in the calculation results including densities corresponding to intra-residue interactions which do not inform us on the protein structure. Conversely, by separating interactions per residue, we can limit the studied interactions to those structuring the α-helix. Figure S4 exemplifies the isosurfaces obtained for a segment of the α-helix.

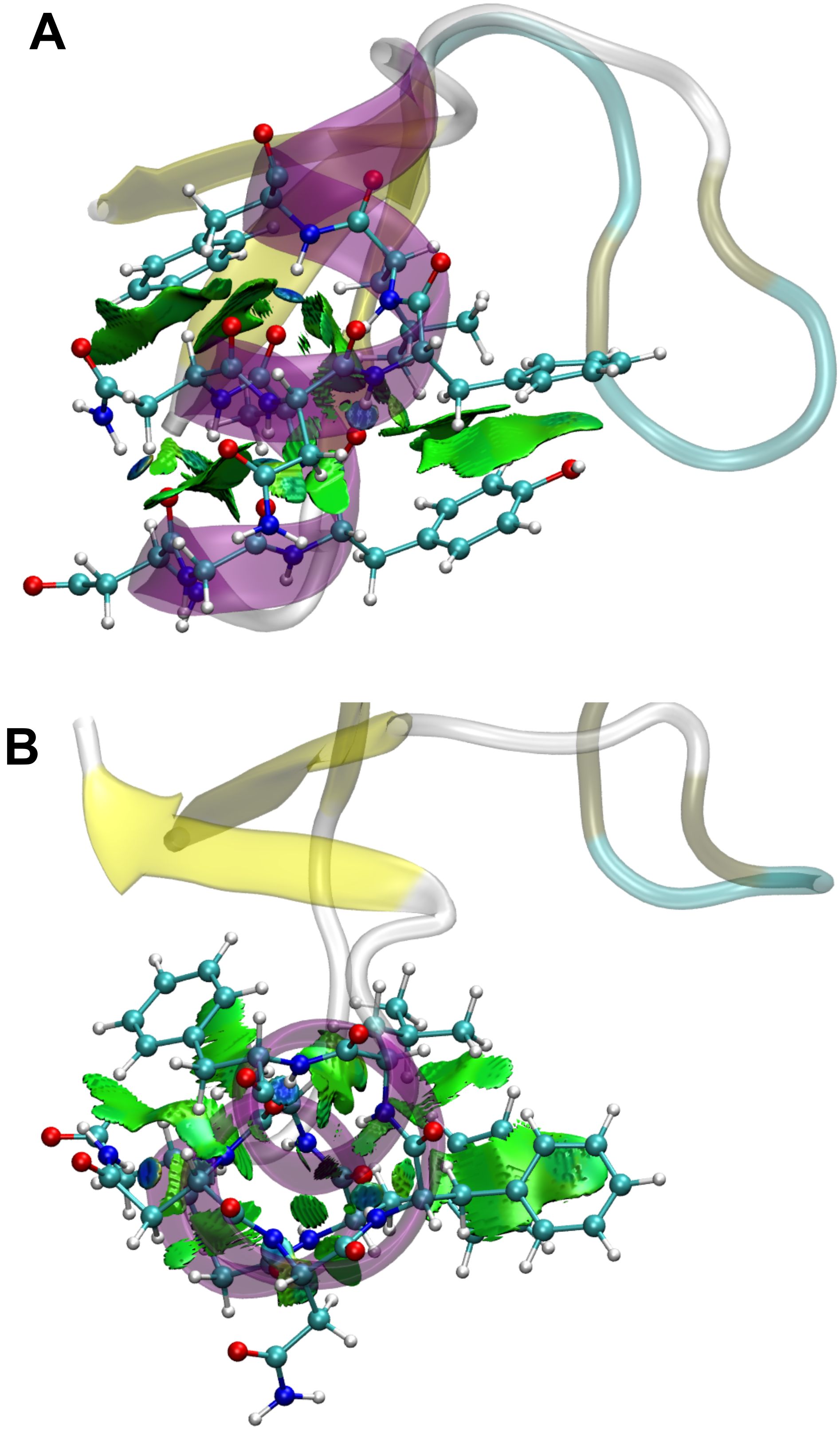

Figure S4: Side (A) and axial (B) views of the alpha helix, showing residues 24Gly to 32Lys along with isosurfaces of the NCI densities between them.

Figure S5 presents the NCI density maps to those obtained exclusively using the inverse inter-residue distances between both all heavy atoms and hydrogen donor and acceptors.

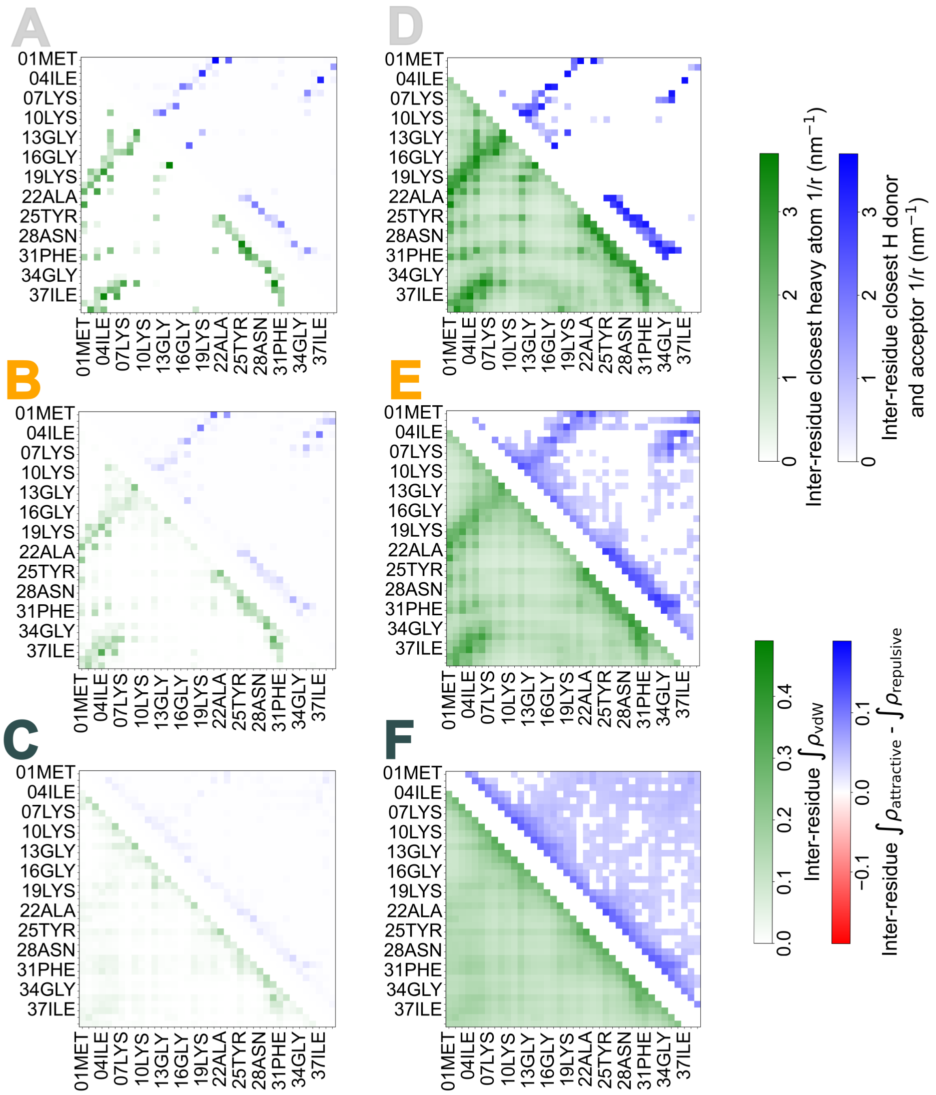

Figure S5: A-C: highest inverse distances among all heavy atoms, in the lower left half of the graph, and among all detected hydrogen bond donors and acceptors, in the higher right half, between each residue pair for the folded, transition and unfolded ensemble respectively. D-F: NCI densities with vdW densities in the lower left half and attractive minus repulsive interactions in the higher right half of the graph for the folded, transition and unfolded ensemble respectively. In all cases only interactions between a residue and those separated by at least three residues have been considered. Results were averaged over 500 random snapshots of each ensemble.

Clustering methodology

Residue pair clustering was carried out using hierarchical agglomerative clustering on a distance matrix obtained by averaging those of each independent transition, since structural motifs may be formed at different time points during independent transitions. The residue pair distance matrix for each transition was obtained using the Move-Split-Merge (MSM) methodology as implemented in the Aeon python package[S1] with a window 0.5, meaning 50% of the trajectory, for the bounding matrix. For each residue pair the 3 NCI density integrals in every sampled frame were inputted treating each integral as independent channels. Each density, per residue pair and transition, was was normalized to have an average of 0 and standard deviation of 1.

Trajectories were clustered using using hierarchical agglomerative clustering on the distance matrix obtained using dynamic time warping (DTW) on the three sums of NCI densities for each characterized structural motif (α1, α2, β12 and β13). The density sums were normalized so their maximum recorded value, considering all transitions, was 1. Unfolding transitions were also reversed for this analysis. To study the optimal number of differentiated pathways, multiple analysis were carried out, namely: elbow method, silhouette analysis, Davis-Bouldin index, PCA and tSNE.

Figure S6 presents the individual normalized densities for each residue pair and average for each determined cluster.

Figure S7 presents the clustering quality metrics used to select the optimal number of clusters for trajectory clustering.

Figures S8, S9 and S10 presents example transitions of each cluster including the total density of each structural motif and the simplified DSSP.

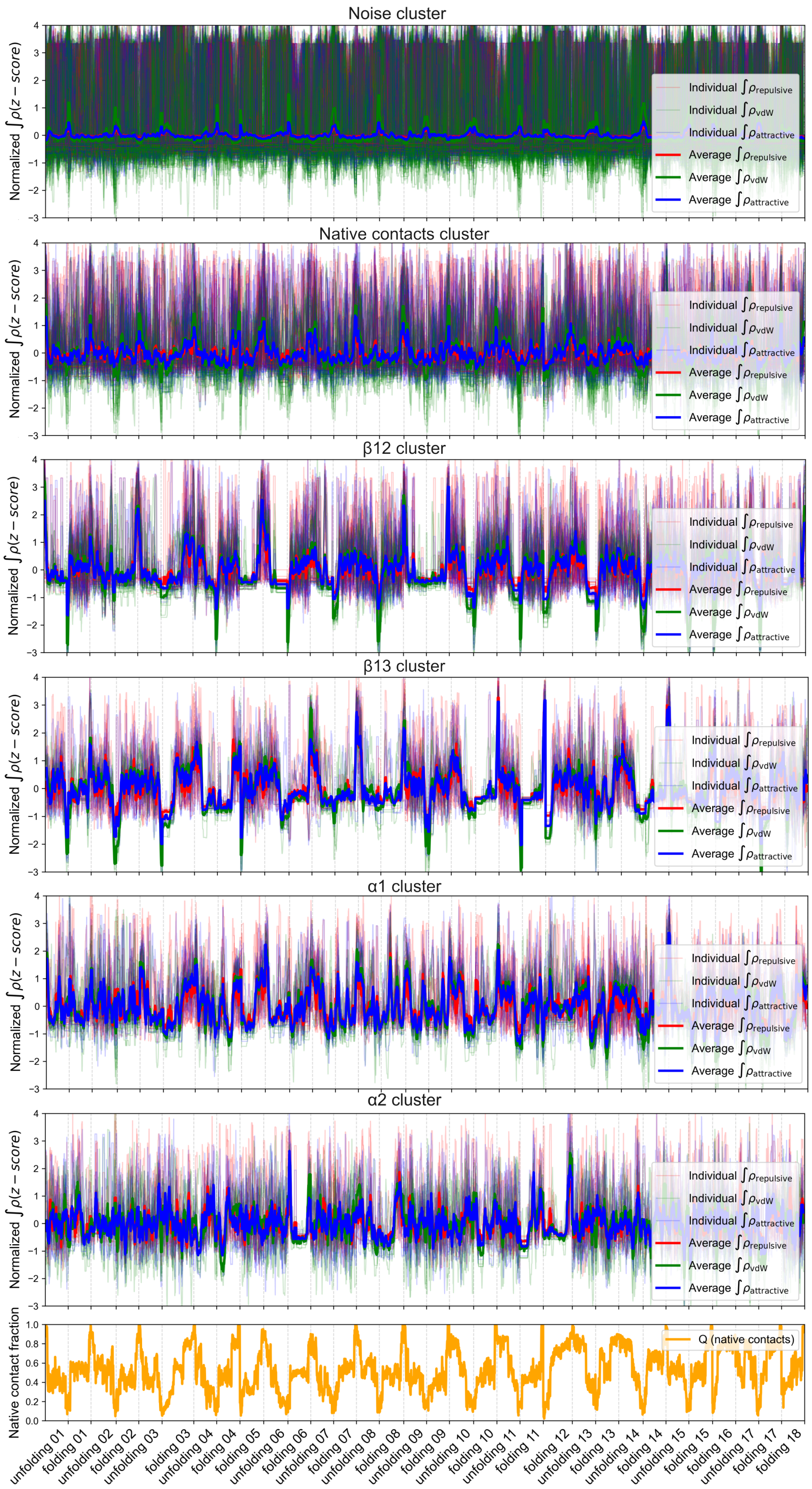

Figure S6: NCI densities for each individual residue pair in a cluster as well as their average for each cluster during all characterized transitions

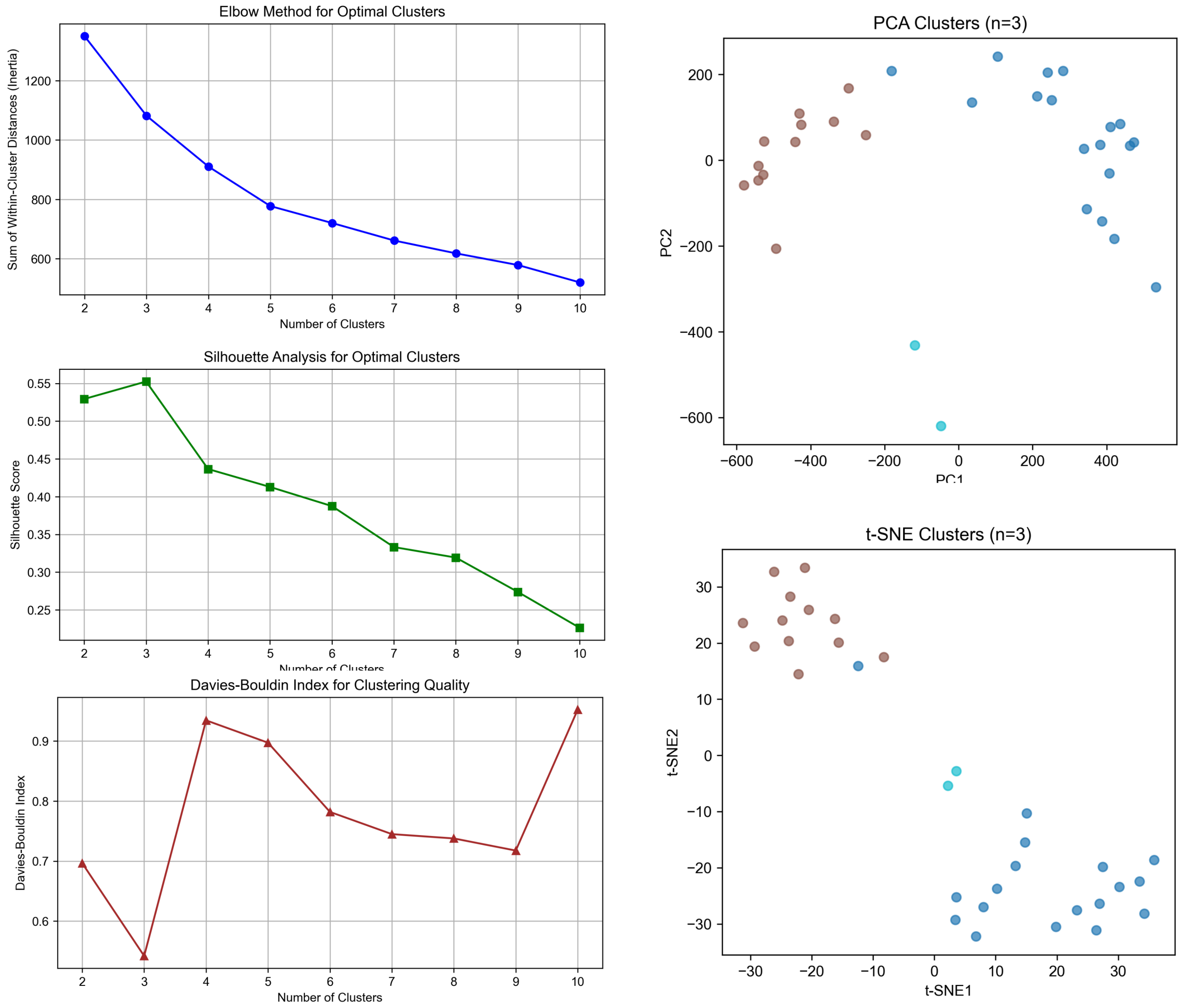

Figure S7: Different clustering quality analyses to determine the number of differentiated pathways

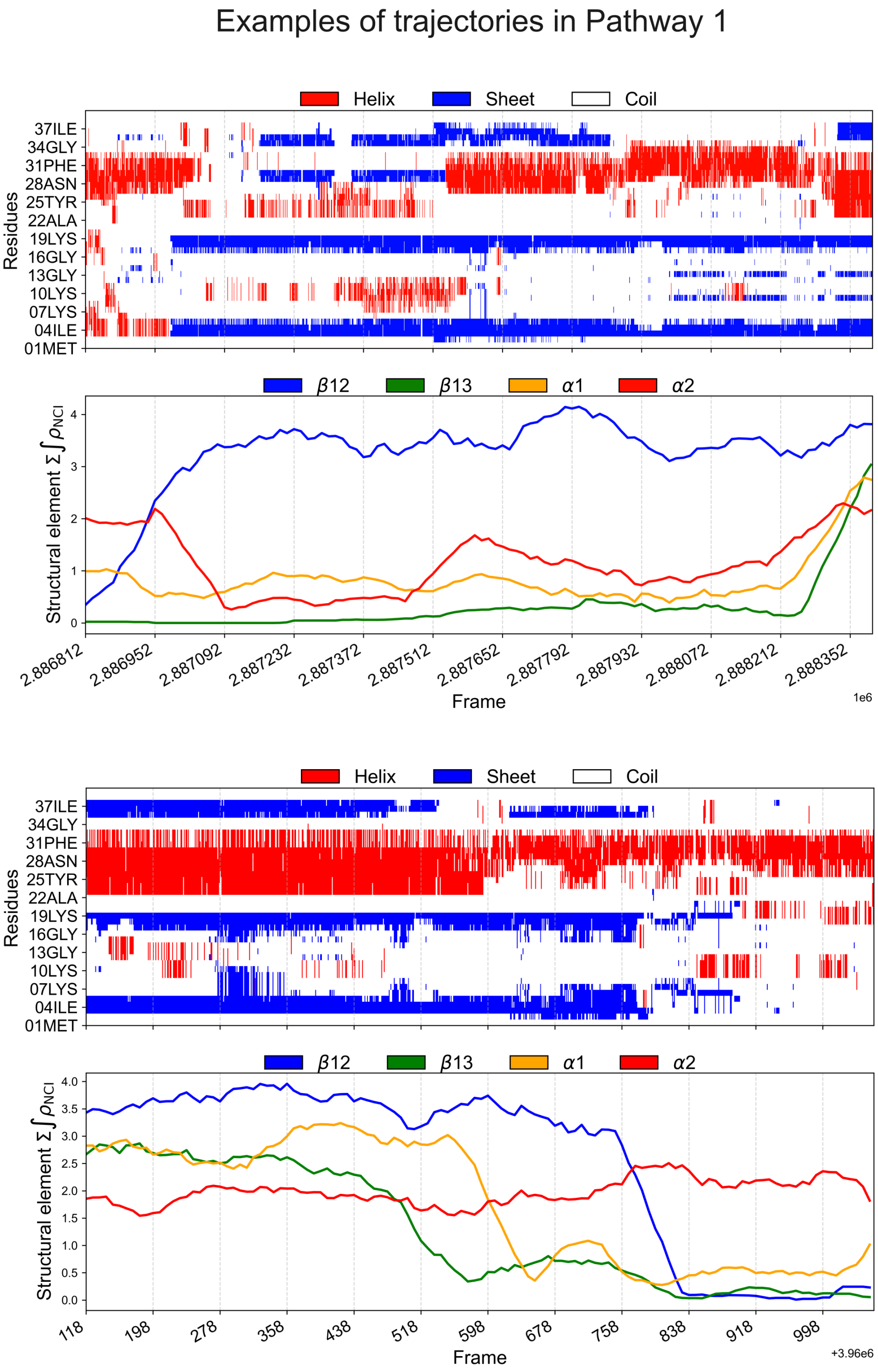

Figure S8: DSSP and sum of NCI density integrals for each characterized structural elements for two example transitions determined to be part of the first pathway via trajectory clustering

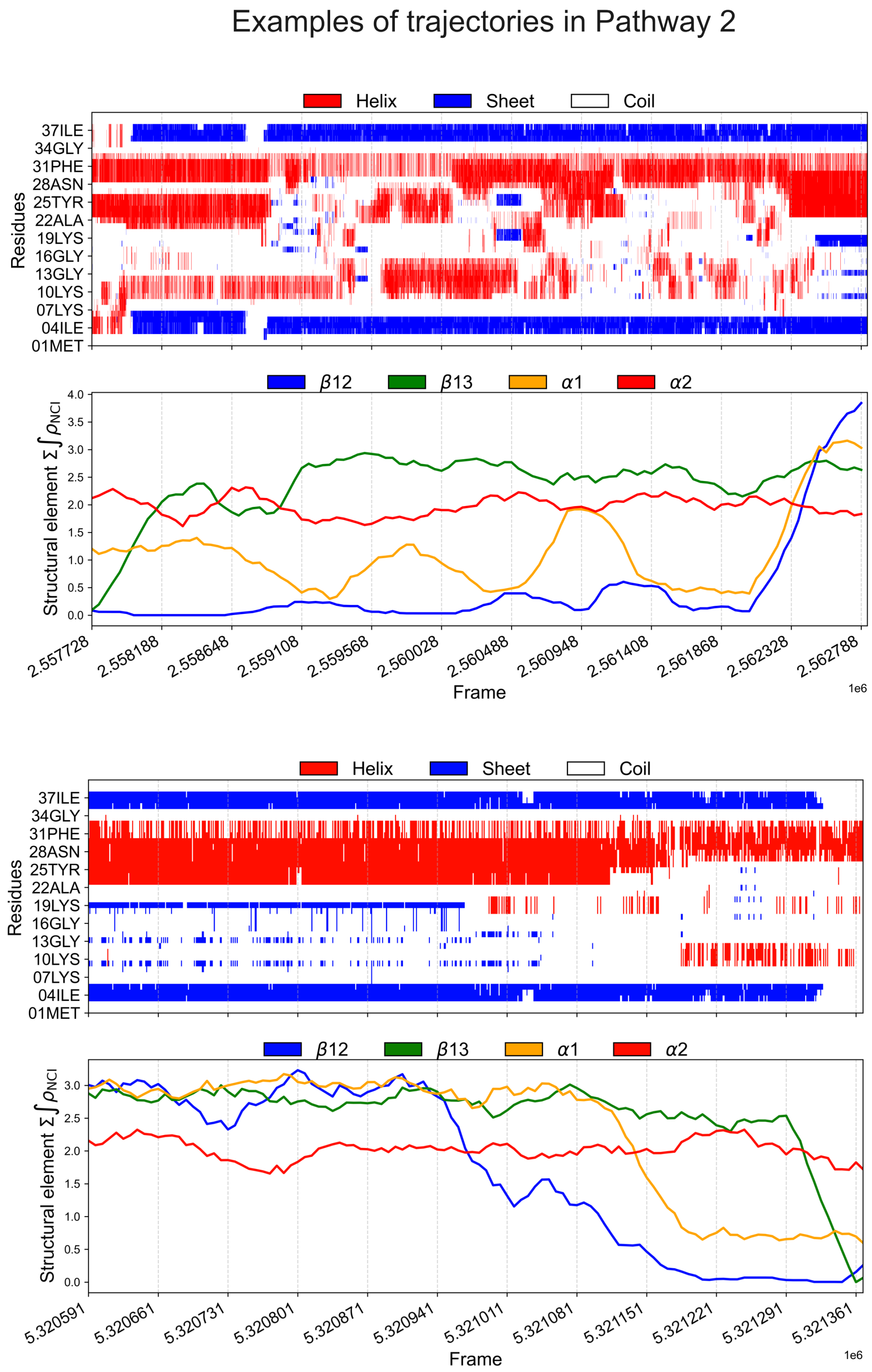

Figure S9: DSSP and sum of NCI density integrals for each characterized structural elements for two example transitions determined to be part of the second pathway via trajectory clustering

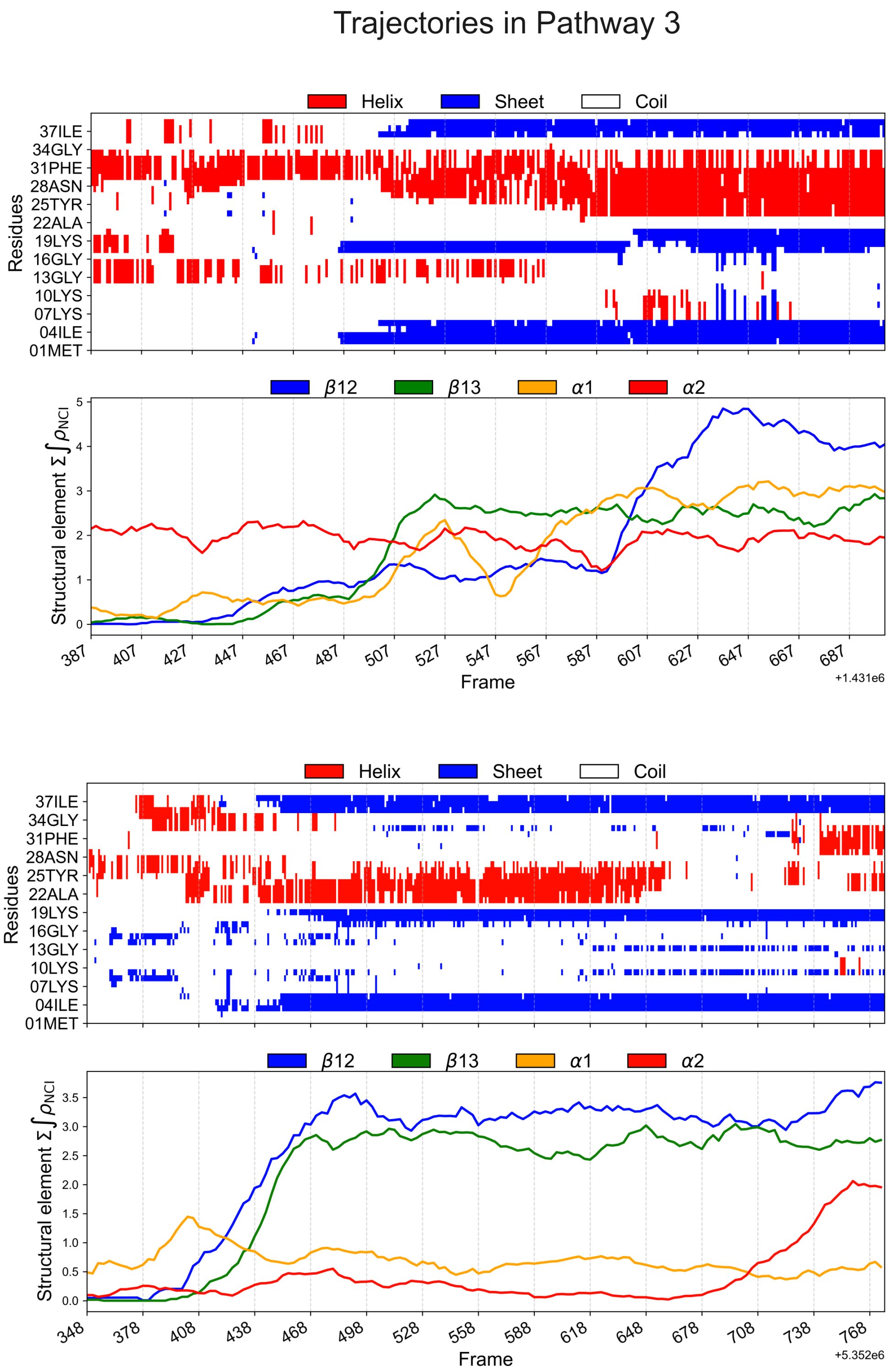

Figure S10: DSSP and sum of NCI density integrals for each characterized structural elements of the two example transitions determined to be part of the third pathway via trajectory clustering

References

(S1) Middlehurst, M.; Ismail-Fawaz, A.; Guillaume, A.; Holder, C.; Guijo-Rubio, D.; Bulatova, G.; Tsaprounis, L.; Mentel, L.; Walter, M.; Schäfer, P.; Bagnall, A. aeon: a Python Toolkit for Learning from Time Series. *J. Mach. Learn. Res.* **2024**, *25*, 1–10.
